# Pre-clinical Safety and Efficacy of Lentiviral Vector-Mediated *Ex Vivo* Stem Cell Gene Therapy for the Treatment of Mucopolysaccharidosis IIIA

**DOI:** 10.1016/j.omtm.2019.04.001

**Published:** 2019-04-06

**Authors:** Stuart M. Ellison, Aiyin Liao, Shaun Wood, Jessica Taylor, Amir Saam Youshani, Sam Rowlston, Helen Parker, Myriam Armant, Alessandra Biffi, Lucas Chan, Farzin Farzaneh, Rob Wynn, Simon A. Jones, Paul Heal, H. Bobby Gaspar, Brian W. Bigger

**Affiliations:** 1 Stem Cell & Neurotherapies Group, School of Biological Sciences, Faculty of Biology Medicine and Health, Division of Cell Matrix Biology and Regenerative Medicine, University of Manchester, Manchester M13 9PT, UK; 2 Boston Children’s Cancer and Blood Disorders Center, Boston Children’s Hospital, Boston, MA 02115, USA; 3 School of Cancer & Pharmaceutical Sciences, King’s College London, Molecular Medicine Group, The Rayne Institute, London SE5 9NT, UK; 4 Manchester Children’s Hospital, NHS Trust, Manchester M13 9WL, UK; 5 Orchard Therapeutics Ltd., London EC4N 6EU, UK; 6 Instititute of Child Health, University College London, London WC1N 1EH, UK

## Abstract

Hematopoietic stem cell gene therapy is a promising therapeutic strategy for the treatment of neurological disorders, since transplanted gene-corrected cells can traffic to the brain, bypassing the blood-brain barrier, to deliver therapeutic protein to the CNS. We have developed this approach for the treatment of Mucopolysaccharidosis type IIIA (MPSIIIA), a devastating lysosomal storage disease that causes progressive cognitive decline, leading to death in early adulthood. In a previous pre-clinical proof-of-concept study, we demonstrated neurological correction of MPSIIIA utilizing hematopoietic stem cell gene therapy via a lentiviral vector encoding the *SGSH* gene. Prior to moving to clinical trial, we have undertaken further studies to evaluate the efficiency of gene transfer into human cells and also safety studies of biodistribution and genotoxicity. Here, we have optimized hCD34^+^ cell transduction with clinical grade SGSH vector to provide improved pharmacodynamics and cell viability and validated effective scale-up and cryopreservation to generate an investigational medicinal product. Utilizing a humanized NSG mouse model, we demonstrate effective engraftment and biodistribution, with no vector shedding or transmission to germline cells. SGSH vector genotoxicity assessment demonstrated low transformation potential, comparable to other lentiviral vectors in the clinic. This data establishes pre-clinical safety and efficacy of HSCGT for MPSIIIA.

## Introduction

Mucopolysaccharidosis type IIIA (MPSIIIA), also known as Sanfilippo syndrome A, is a severe, progressive, neurodegenerative disorder caused by loss-of-function mutations in the N-sulfoglucosamine sulfohydrolase (*SGSH*) gene.^1^ SGSH is one of several essential lysosomal enzymes involved in the sequential degradation of the glycosaminoglycan (GAG) heparan sulfate (HS).^1^ Absence of functional SGSH leads to the abnormal accumulation of partially degraded, highly sulphated HS in cells throughout the body, resulting in cellular toxicity and impaired organ function. The brain is predominantly affected, and clinical symptoms, which are typically diagnosed at 2 to 4 years of age, include cognitive delay, neurodegeneration, and severe behavioral disturbances.^2^ In addition, patients also suffer from non-neurological symptoms, including hepato- and splenomegaly, digestive tract problems, speech delay, hirsutism, recurrent ear, nose, and throat infections, and facial dysmorphisms.^3^ Disease pathology progressively worsens over time, and affected individuals do not typically survive beyond late teenage years or early adulthood.

Autologous hematopoietic stem cell gene therapy (HSCGT) is an appealing therapeutic strategy for the treatment of neurodegenerative lysosomal storage disorders, since patients’ own hematopoietic progeny cells can be genetically engineered to express or overexpress functional lysosomal enzyme. Modified cells, especially of the macrophage lineage, can migrate to affected tissues and importantly possess the ability to pass through the blood-brain barrier (BBB), engraft in the CNS, and secrete therapeutic enzyme that can be taken up by affected neurons, thereby enabling the breakdown of the stored material and cross-correction of afflicted cells in the CNS as well as in the periphery.^4^ Crucially, by increasing the level of functional gene replacement per genetically modified cells within hematopoietic stem cells (HSCs) and their progeny, there is an opportunity for supra-normal enzyme expression, thereby enhancing efficacy compared to HSC transplant alone.^5^, ^6^ The intrinsic self-renewal ability of HSCs means that HSCGT has the potential to be a one-off treatment without the need for repeated dosing, as is the case with current enzyme-replacement therapies. Encouragingly, the HSCGT approach has been successfully evaluated for the similar metabolic storage disorder metachromatic leukodystrophy (MLD) and also for the peroxisomal condition CCALD (childhood cerebral adreno-leukodystrophy) and the primary immunodeficiency disorders adenosine deaminase severe combined immunodeficiency (ADA-SCID) and Wiskott-Aldrich syndrome (WAS), demonstrating promising clinical outcomes.^4^, ^7^, ^8^, ^9^, ^10^, ^11^

Alternative MPSIIIA treatment methodologies currently being evaluated include repeated intracerebrospinal fluid infusions of recombinant human SGSH (rhSGSH) (https://clinicaltrials.gov/; NCT #01155778, #01299727, #02060526, #02350816),^12^, ^13^, ^14^, ^15^ direct injection into the brain with AAV vector encoding SGSH and SUMF1 (lysogene SAF 301, NCT #01474343),^16^, ^17^, ^18^ and intravenous delivery of CNS targeting AAV9 vector (scAAV9.U1a.hSGSH) (Abeona ABO-102, NCT #02716246).^19^ In addition, a phase I-II clinical trial using an AAV9 vector containing hSGSH for intracerebroventricular (ICV) delivery (Esteve-EGT-101) has recently opened and are currently enrolling patients. Completed MPSIIIA trials to date have demonstrated limited efficacy in early evaluations, and in some cases high numbers of severe adverse events have been reported.^12^ Long-term clinical data from Abeona ABO-102 (NCT #02716246) is still awaited. AAV gene therapy treatments delivered directly to the brain are often highly invasive, while systemically delivered AAV vectors can be costly to scale up, with the potential to generate undesirable immune responses limiting efficacy of the treatment.^20^ Furthermore, a number patients display pre-existing immunity to AAV serotypes with CNS tropism, limiting the potential scope of treatment.

We have developed the HSCGT approach for the treatment of MPSIIIA based on lentiviral-mediated transfer of the *SGSH* gene under the control of the CD11b promoter to target gene expression to myeloid cells trafficking to the brain. In a pre-clinical proof-of-concept study, we previously demonstrated disease correction following transplantation of gene-corrected autologous SGSH-deficient murine HSCs into busulfan-conditioned MPSIIIA mice.^21^ Transduction of autologous MPSIIIA HSCs with CD11b.SGSH lentiviral vector (LV) normalized the hyperactivity characteristics of the disease, brain HS, secondary storage, lysosomal compartment size, and neuroinflammation in MPSIIIA mice, whereas a phosphoglycerate kinase mammalian promoter (PGK)-driven vector could only mediate partial correction in many of these parameters. Increased SGSH expression from myeloid-derived cells migrating into the brain and differentiating into microglia-like cells resulted in improved brain enzyme without changing peripheral enzyme overexpression, making the CD11b vector more target specific for the brain.^21^

Following successful proof of concept in the MPSIIIA mouse model, here we demonstrate the safety and efficacy of clinical grade GMP CD11b.SGSH lentiviral vector prior to a first in human clinical trial in accordance with regulatory guidelines, evaluating vector batch equivalence, optimal dosing, transduction scale-up and cryopreservation, engraftment, biodistribution, systemic toxicity, and vector genotoxicity.

## Results

### GMP CD11b.SGSH LV Is Equivalent to Research Grade LV: *In Vivo* Vector-Bridging Study

**Figure 1:**
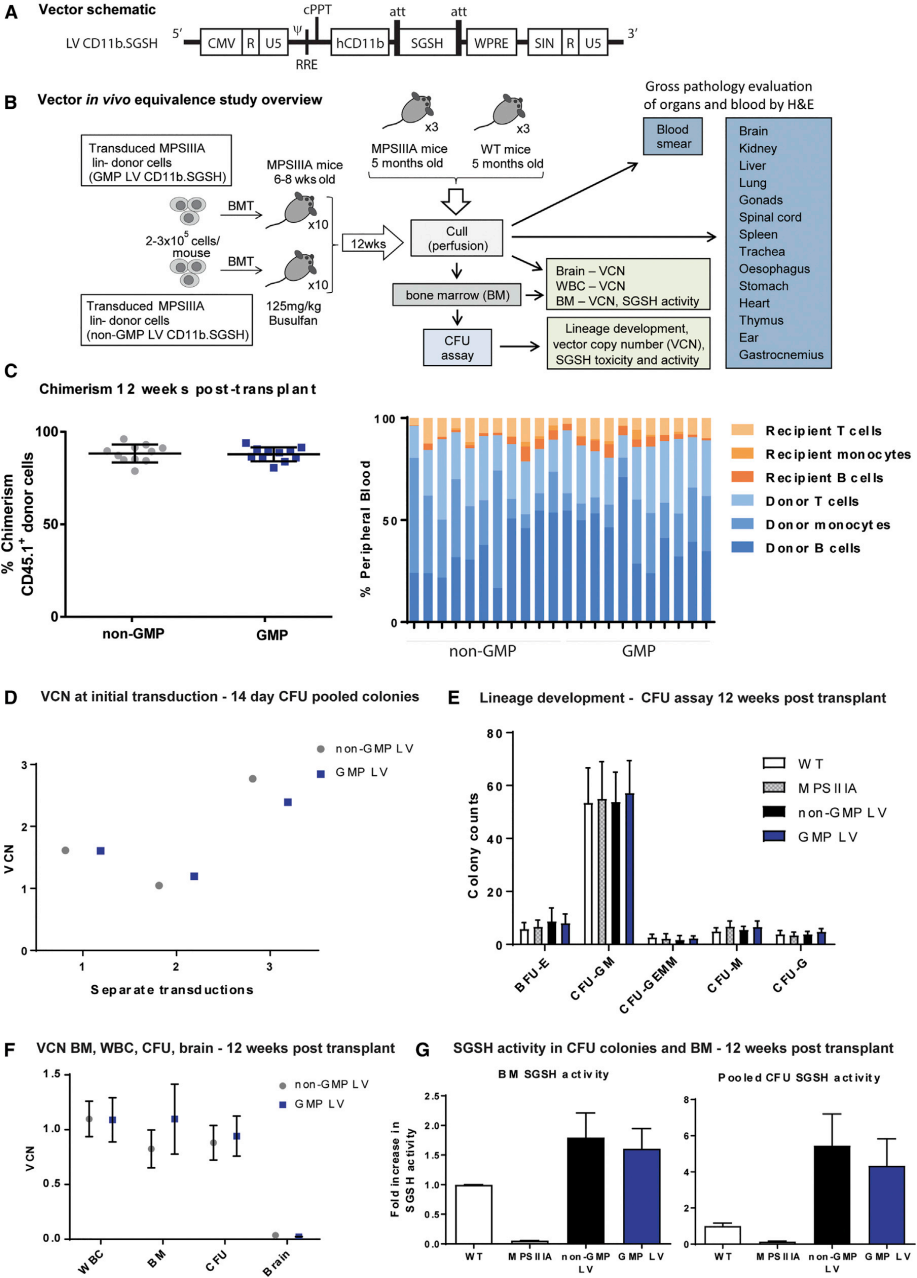
GMP LV CD11b.SGSH Is Equivalent to Its Research Grade Counterpart *In Vivo* (A) Vector design. pCCL LV containing a human myeloid CD11b promoter driving expression of codon-optimized SGSH. (B) MPSIIIA bone marrow was lineage depleted and transduced with GMP or non-GMP CD11b.SGSH LV at an MOI of 60, then transplanted into busulfan-myeloablated MPSIIIA mice. (C) Twelve weeks post-transplantation, the donor chimerism in WBCs was determined by flow cytometry (n = 10 per group). (D) VCN was determined for each transplant batch at initial transduction following 14 days in methylcellulose culture. (E) Hematopoietic lineage development was assessed by CFU assay 12 weeks post-transplant. CFU-granulocyte, macrophage (CFU-GM); BFU-erythroid (BFU-E); CFU-granulocyte, erythroid, macrophage, megakaryocyte (CFU-GEMM); CFU-granulocyte (CFU-G); and CFU-macrophage (CFU-M). (F) VCN in white blood cells (WBCs), bone marrow (BM), pooled CFU colonies, and the brain was assessed 12 weeks post-transplant. (G) SGSH enzyme activity was assessed in pooled CFU colonies and BM 12 weeks post-transplant (one-way ANOVA Kruskal-Wallis test).

To develop HSCGT for MPSIIIA patients, we produced a third-generation self-inactivating (SIN) LV with a codon optimized SGSH transgene driven by the myeloid-specific CD11b promoter (CD11b.SGSH LV), manufactured to good manufacturing practice (GMP) standard (Figure 1A).^21^ In order to demonstrate that GMP vector has the comparable efficacy and safety profile as research-grade (non-GMP) vector (as used in earlier pre-clinical proof-of-concept studies^21^), we devised a short-term *in vivo* bridging study (Figure 1B). MPSIIIA recipient mice (CD45.2+ve) were transplanted with either GMP- or non-GMP LV-transduced MPSIIIA lineage-depleted progenitor donor cells (CD45.1+ve) and evaluated at 12 weeks post-transplant (Figure 1B). Mean donor cell engraftment for both the GMP and non-GMP-transduced groups was 87.9% and 88.3%, respectively (Figure 1C). Flow cytometry analysis of blood highlighted some variation in leucocyte composition in individual mice; however, overall, comparable proportions of donor and recipient B cells (CD19^+^), T cells (CD3^+^), and monocytes (CD11b^+^) were observed between the GMP and non-GMP groups (Figure 1C). Transplants were performed in separate batches as donor and recipient mice became available, with an equal number of GMP and non-GMP LV-transplanted mice in each batch. There was no difference in transduction efficiency between vector grades in terms of vector copy numbers (VCNs); however, variation in integrated VCNs was observed between different transplant batches, likely due to differences between donor hematopoietic stem-cell-enriched cell lots (Figure 1D).

At harvest, a proportion of bone marrow was seeded for colony-forming unit (CFU) assay to assess normal lineage development. Similar numbers of colony types were observed among wild-type (WT), MPSIIIA, GMP, and non-GMP LV-transplanted groups, suggesting no disruption in stem cell capacity following genetic modification (Figure 1E). The VCN was assessed in white blood cells (WBCs), bone marrow (BM), CFU colonies, and the brain of transplanted mice, with no differences observed between GMP and non-GMP groups (Figure 1F). An increase in SGSH enzyme activity was detected in the BM and pooled CFU colonies for both GMP and non-GMP groups compared to WT and MPSIIIA controls, with no significant difference between the two. SGSH activity was also measured in the brain and plasma; however, no increase was observed compared to non-transplanted MPSIIIA controls after 12 weeks (S.M.E., data not shown). In the proof-of-concept study performed previously, 11% of wild-type SGSH activity was achieved in the brain of treated MPSIIIA mice 6 months post-transplant, this suggests 12 weeks may only allow partial monocyte migration to the brain, insufficient to achieve significant increases in SGSH activity.^21^ This HSGCT bridging study confirms clinical GMP-grade SGSH LV is equivalent to its non-GMP research-grade counterpart in MPSIIIA mice.

### CD11b.SGSH LV-Mediated HSCGT Reduces Kidney and Myocardial Vacuolation in MPSIIIA Mice

A toxicology study was performed on tissues harvested in the vector-bridging study from GMP and non-GMP LV transplant group animals and compared to untreated MPSIIIA mice. Key histopathology findings were present in kidney, heart, and liver (Table S1). Vacuolation of the tubular epithelium of the distal tubules of the cortex in the kidney and myocardial vacuolation was seen in both untreated and treated animals, with the treated animals showing a reduction in the severity of the finding over untreated MPSIIIA controls. Decreased glycogen was observed in the livers of occasional MPSIIIA mice given cells transduced by non-GMP and GMP LV (3/10 and 4/10, respectively).

Another finding in the liver included minimal centrilobular hypertrophy (3/10) and minimal to slight focal necrosis of the hepatocytes (3/10). These findings were only seen in MPSIIIA animals given cells transduced with GMP LV and were accompanied with an increased incidence of minimal to slight inflammatory cell infiltrates in the liver of these animals (6/10) when compared to all other groups. This finding was also present at a low incidence for the non-GMP LV-treated animals (1/10). It is considered that the focal necrosis and increased incidence of inflammatory infiltrates were likely sequelae of the earlier myeloablative conditioning, with the incidence in the affected animals being completely random and not due to the vector grade. Myeloablative conditioning was achieved with the use of busulfan, the formulation of which is known to be hepatotoxic, causing lesions similar to those observed here.

### CD11b LV Effectively Transduces Human Hematopoietic Stem Cells

**Figure 2:**
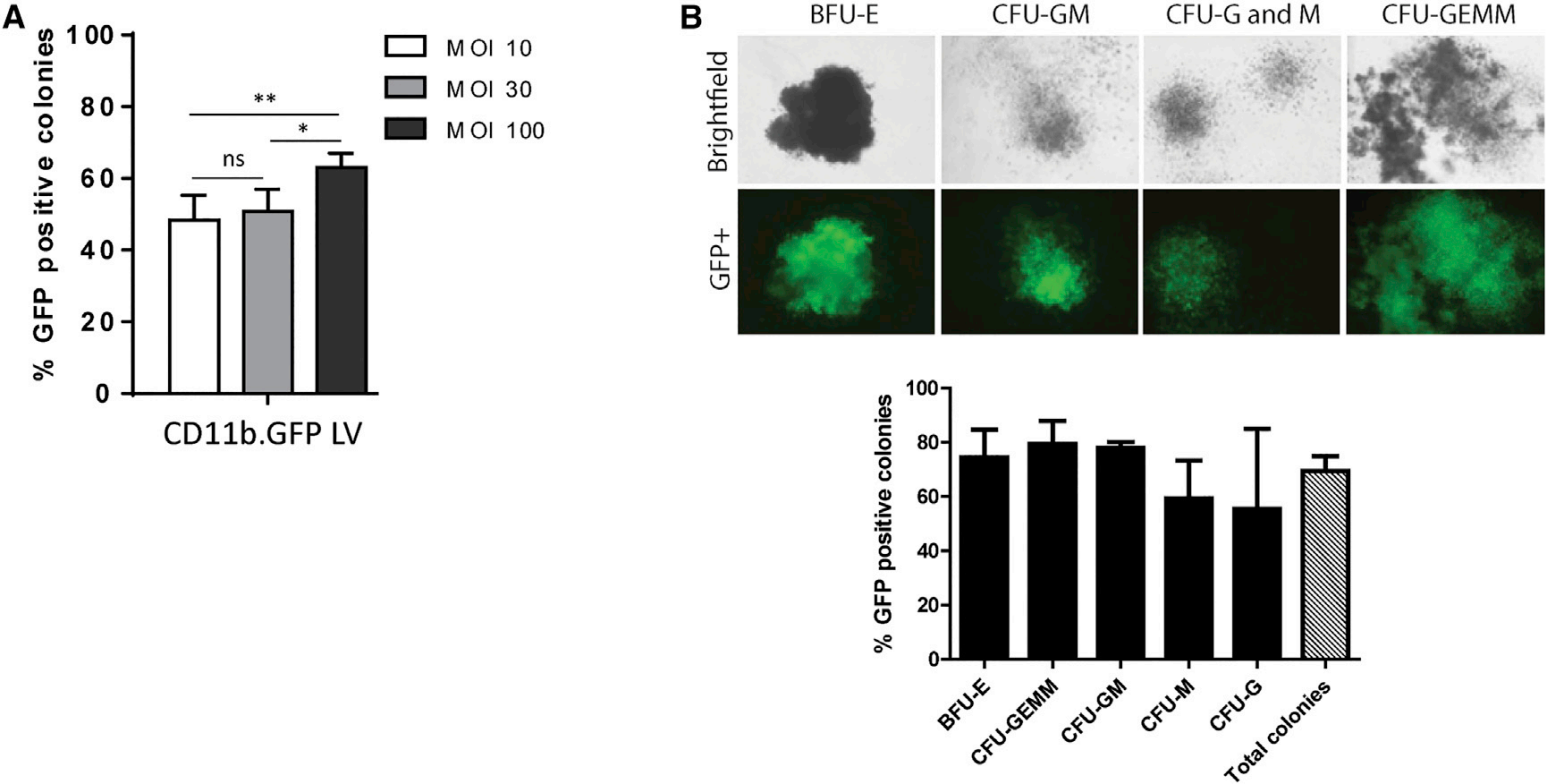
Characterization of Human CD34^+^ Cell Transduction with CD11b.GFP LV (A) Umbilical cord blood (UCB) hCD34^+^ cells were transduced with an increasing MOI of CD11b.GFP LV and transduction efficiency determined by counting GFP-positive colonies following colony-forming unit (CFU) assay (*p < 0.05, **p < 0.01). (B) hCD34^+^ cells were transduced with CD11b.GFP LV at an MOI of 100 and the transduction efficiency evaluated in BFU-E, CFU-GEMM, CFU-GM, CFU-M, and CFU-G progenitors. Representative images of CD11b.GFP LV transduced progenitors.

In order to investigate transduction efficiency of human hematopoietic stem cells with the CD11b-driven LV, we first utilized a LV expressing GFP (CD11b.GFP LV) to allow simple visualization of transduced cell progeny. Umbilical-cord-blood-sourced CD34^+^ cells were transduced at an increasing MOI range of 10, 30, and 100, with an MOI of 100 used in several completed clinical studies.^4^, ^22^ Transduced CD34^+^ cells were plated in methylcellulose culture for 14 days, and the percentage of GFP +ve cells for each LV dose was determined. As expected, an MOI of 100 provided the highest transduction efficiency of 63%, significantly higher than MOIs of 10 and 30 (Figure 2A). We further evaluated the pattern of transduction for different hematopoietic progenitor types and observed similar numbers of transduced progenitors (Figure 2B).

**Figure 3:**
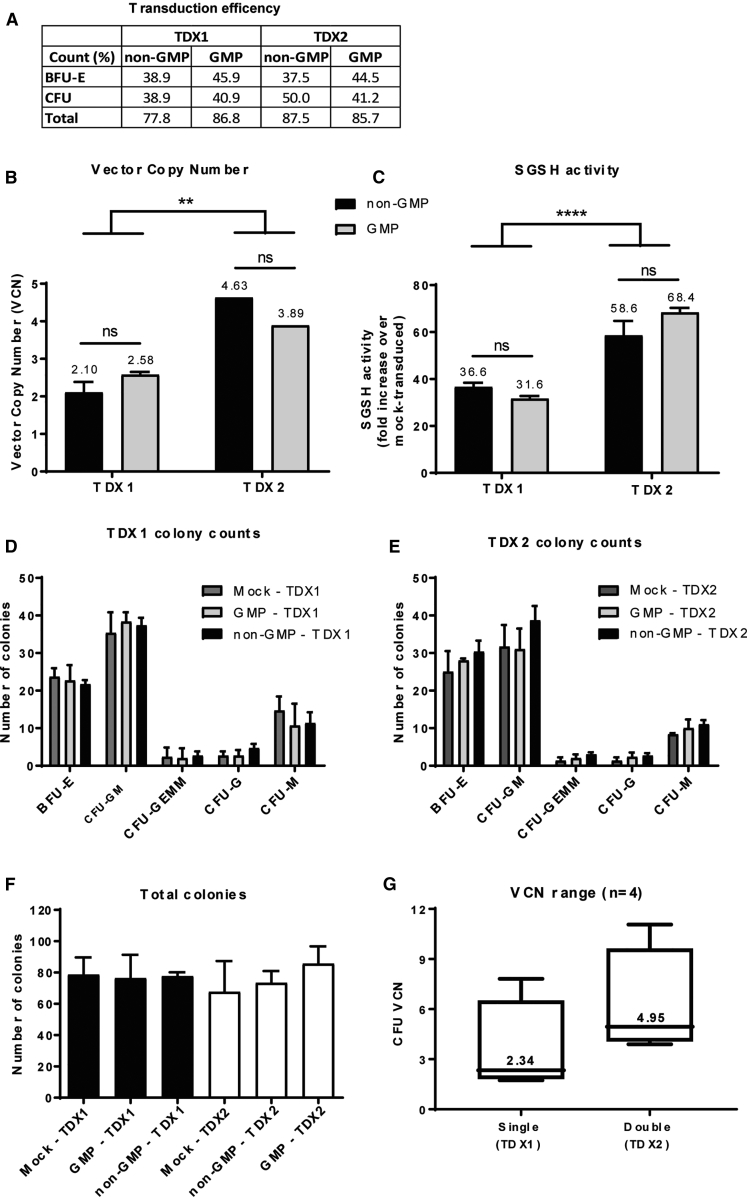
CD11b.SGSH LV Equivalence Testing and Dose Optimization in CD34^+^ Cells hCD34^+^ cells were transduced with GMP or non-GMP LV at an MOI of 100 and evaluated in the CFU assay. Single (TDX1) versus double (TDX2) transductions were compared. (A) Transduction efficiency determined by qPCR assessment of individual colonies and calculating the percentage of positive colonies containing integrated transgene (n = 16 BFU-E; n = 16 CFU colonies). (B) VCN determined in pooled CFU colonies by qPCR (**p < 0.005). (C) SGSH enzyme activity in pooled CFU colonies (****p < 0.0001). (D) CFU progenitor counts following TDX1 or TDX2. (E) CFU progenitor counts following TDX2. (F) Total CFU colony counts. (G) The experiment was repeated four times with different batches of CD34^+^ cells and the VCN range for TDX1s and TDX2s evaluated.

An effective HSCGT requires sufficient HSC genetic manipulation to provide a therapeutic level of disease correction without impairing normal stem cell expansion or causing adverse effects. We therefore evaluated the optimal transduction dose of our therapeutic CD11b.SGSH LV in human CD34^+^ cells. Experiments were designed to both evaluate single versus double transduction and to confirm equivalence between GMP- and non-GMP-grade LV batches *in vitro*. Mock-transduced cells undergoing the same manipulations as transduced cells but without culture in the presence of lentiviral vector were included as controls. Overall transduction efficiency was high following both single transduction (TDX1) and double transduction (TDX2) with both vector grades and with similar proportions of erythroid burst-forming unit (BFU-E) and colony-forming unit (CFU) colonies transduced (Figure 3A). TDX1 resulted in VCNs in pooled CFU colonies of 2.58 and 2.1 for GMP and non-GMP, respectively, compared to 3.89 (GMP) and 4.63 (non-GMP) for TDX2 (Figure 3B). There was no change in VCN between vector grades; however, a statistically significant difference was observed between single and double-transduced CD34^+^ cells. SGSH enzyme activity also improved considerably following a double transduction with either vector grade giving a 68.4 (GMP) and 58.6 (non-GMP) fold increase in SGSH enzyme activity over mock-transduced cells compared to a 31.6 (GMP) and 36.6 (non-GMP) fold increase following a single transduction (Figure 3C). Furthermore, there was no significant difference in SGSH activity between GMP and non-GMP transduced groups, confirming vector comparability. The distribution of hematopoietic progenitors for all groups also followed a normal pattern indicating no lineage skewing (Figures 3D and 3E). The yield of cells recovered after 14 days in CFU culture from those cells incubated for 48 hours (TDX2 mock-transduced) was equivalent to cells incubated for 24 hours (TDX1 mock-transduced cells) (Figure 3F). Transduction experiments were repeated four times in total, with CD34^+^ cells sourced from either umbilical cord blood (UCB) or mobilized peripheral blood (MPB) (Figure 3G and data not shown). Achievable VCNs from a single transduction of hCD34^+^ cells ranged from 1.73 to 7.81 with a median of 2.34 compared to a double transduction, where the VCN ranged from 3.89 to 11.06 with a median of 4.95 (Figure 3G).

### Effective Scale-Up of hCD34^+^ Cell Transduction and Cryopreservation

In the clinical setting, a dose in the region of > 5 × 10^6^ transduced CD34^+^ cells/kg will be needed in order to treat patients.^4^ Furthermore, transduced cells will require cryopreservation in order to provide sufficient time for shipment of drug product to the local hospital and conditioning of the patient after drug product release prior to transplant. We performed a large-scale CD34^+^ cell transduction validation run, using GMP grade LV under GMP-like conditions including a cryopreservation step of ≥ 6 weeks (52 days) to replicate manufacture of an investigational medicinal product (IMP) and supporting stability of cryopreserved formulation of drug product. HSCs were isolated from a MPB apheresis unit by immuno-magnetic CD34^+^ cell selection. An initial pre-stimulation step was performed with 60 × 10^6^ CD34^+^ cells followed by two rounds of LV transduction at an MOI of 100 in accordance with the likely future clinical transduction protocol to achieve maximal levels of CD34^+^ cell transduction and integrated vector copies. Mock-transduced CD34^+^ cells were included as controls.

**Figure 4:**
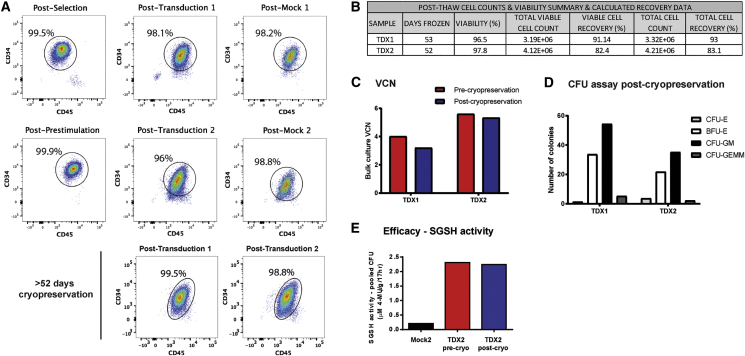
CD34^+^ Cell Transductions Scaled Up and Cryopreservation Validated for Clinical Application (A) CD34^+^ cell purity and viability accessed by flow cytometry at post-selection, post-prestimulation, post-transduction, and post-cryopreservation. (B) Post-thaw cell counts, viability, and total cell percentage recovery at 52 days after cryopreservation. (C) VCN of bulk liquid culture transduced cells pre- and post-cryopreservation for TDX1 and TDX2 cell groups. (D) The CFU assay was performed post-cryopreservation and the number of BFU-E and CFU colonies determined for TDX1 and TDX2 groups. (E) Evaluation of SGSH activity in transduced cells pre- and post-cryopreservation.

The percentage of viable CD34^+^CD45^+^ cells post-selection, pre-transduction, post-TDX1, post-TDX2, and post-mock-transduction (both single and double) were all 96% or above (Figure 4A). Furthermore, cryopreservation of cells post-TDX1 or -TDX2 had no adverse effect on cell viability, with 99.5% and 98.8% viable CD34^+^CD45^+^ cells recorded, respectively. Post-thaw cell counts and viability (trypan blue staining) for TDX1 and TDX2 samples are summarized in Figure 4B. Viable cell recovery post-thaw was 91.1% for TDX1 and 82.4% for TDX2. A double transduction at large scale achieved a higher VCN than a single transduction, 5.6 (TDX2) versus 4.02 (TDX1), respectively (Figure 4C), comparable to VCNs achieved at small scale (Figure 3F). Post-cryopreservation, similar VCNs of 5.33 (TDX2) and 3.2 (TDX1) were recorded. A similar pattern of CFU and BFU-E progenitors was observed for both TDX1 and TDX2 post-cryopreservation in the CFU assay (Figure 4D); however, we observed a drop in total number of colonies recovered in the TDX2 group compared to TDX1 (62.8 versus 94.5 total colonies, respectively). SGSH enzyme activity remained the same in transduced cells (TDX2 group) before and after cryopreservation (Figure 4E). The large-scale validation run demonstrates that transduction of CD34^+^ cells with GMP CD11b.SGSH LV can be effectively scaled up for the clinic and that 52 days cryopreservation has no detrimental effect on efficacy or cell viability.

### Normal Engraftment and Biodistribution of SGSH LV-Transduced CD34^+^ Cells in a Humanized Mouse Model

**Figure 5:**
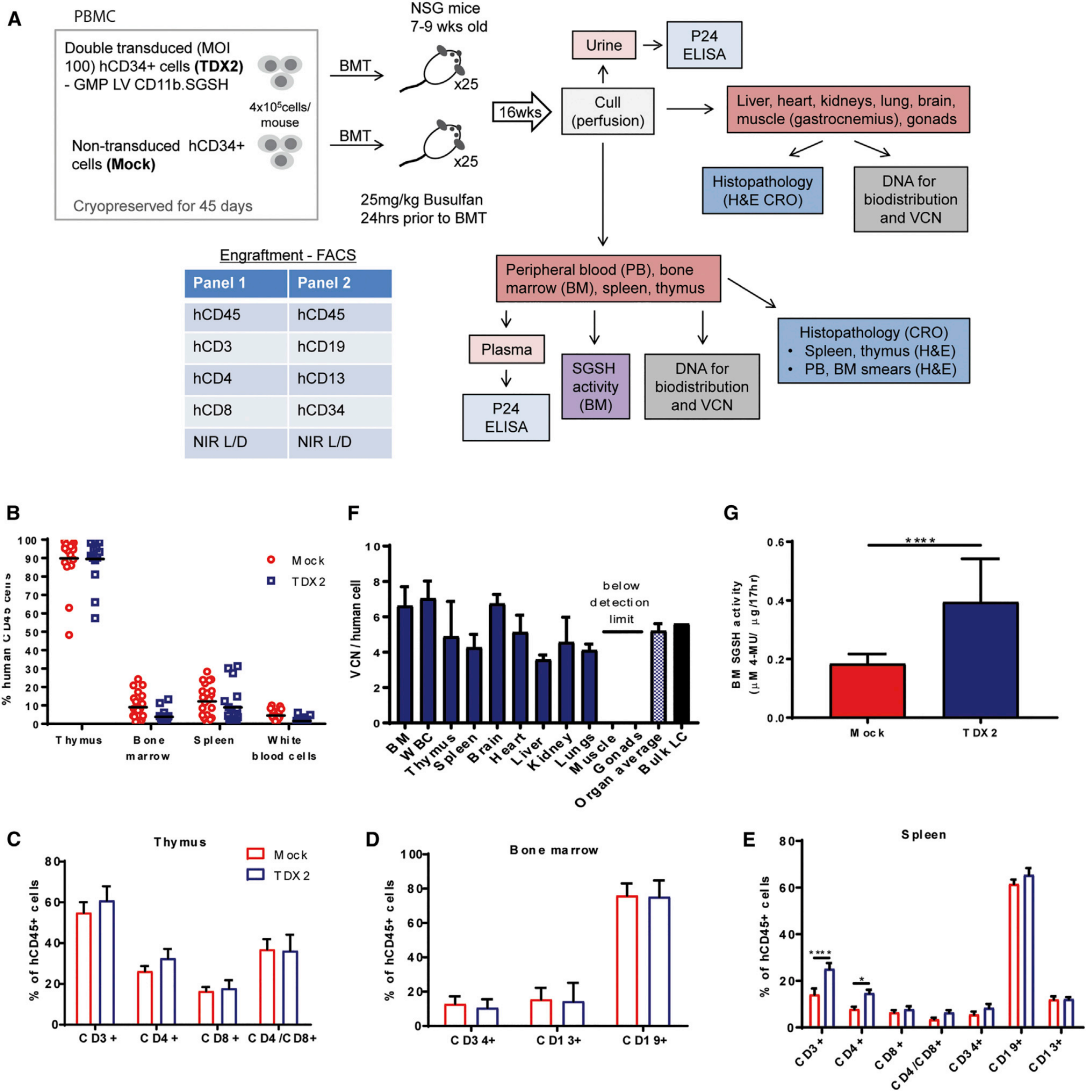
NSG Engraftment and Biodistribution Study (A) NSG engraftment and biodistribution study design. (B) Human leucocyte (CD45^+^ cells) engraftment in the hematopoietic organs of NSG mice transplanted with TDX2 or mock-transduced, 52 day cryopreserved human CD34^+^ cells from PBMCs. (C) Human T cell populations in the thymus of transplanted mice. (D) Stem cell (CD34^+^), myeloid cell (CD13^+^), and B cell (CD19^+^) populations in the BM of transplanted mice. (E) T cell (CD3^+^, CD4^+^, CD8^+^), stem cell (CD34^+^), myeloid cell (CD13^+^), and B cell (CD19^+^) populations in the spleens of transplanted mice (*p <0.05, ****p < 0.001). (F) VCN per human cell was determined in the BM, WBCs, thymus, spleen, brain, heart, liver, kidney, lungs, gastrocnemius muscle, and gonads of TDX2 transplanted mice by qPCR. VCN was also determined for transduced cells before transplant (bulk LC [liquid culture]) (G). SGSH enzyme activity in the BM of mock- and TDX2 transplanted NSG mice (****p < 0.0001).

An engraftment and biodistribution study was performed to evaluate if transduction of human CD34^+^ cells with GMP SGSH LV affects their ability to engraft, differentiate, and distribute to hematopoietic organs in a humanized mouse model. Although HSCs sourced from umbilical cord blood demonstrate greater engraftment potential in NOD.Cg-Prkdcscid Il2rgtm1Wjl/SzJ (NSG) mice^23^ (Figure S1), HSCs isolated from mobilized peripheral blood were chosen as the preferred source, since they more closely represent the autologous cells to be used in the clinic. Here, we utilized the 52 day cryopreserved, TDX2 and mock-transduced (MOCK) CD34^+^ cells from the large-scale transduction validation run. An overview of the study design is depicted in Figure 5A. Mice were treated with a partial myeloablative dose of 25 mg/kg busulfan (Bu) 24 h prior to transplant.

Engraftment of human leukocytes and differentiation into specific lineages was evaluated in the hematopoietic organs (BM, spleen, and thymus) and peripheral blood (PB) of NSG mice 16 weeks post-transplant (Figure 5B). The thymus demonstrated the highest level of human cell engraftment, with average hCD45^+^ cell engraftment levels of 89.9% and 89.5% for the mock and TDX2 groups, respectively (Figure 5B). In the BM, engraftment was 9.0% and 3.8%, in the spleen 12.2% and 8.9%, and in the WBCs 4.5% and 1.5% for the mock and TDX2 groups, respectively. There was no significant difference in hCD45^+^ cell engraftment between mock and TDX2 groups in the individual organs tested (two-way ANOVA multiple-comparison post-hoc Tukey test). We further investigated the leucocyte compositions in the hematopoietic organs. In the thymus, the primary lymphoid organ of the immune system where T cells mature, there were equivalent proportions of CD3^+^, CD4^+^, CD8^+^, and CD4+CD8^+^ cells between the mock and TDX2 treatment groups (two-way ANOVA multiple comparison test) (Figure 5C). In the BM, we observed equivalent stem cell (CD34^+^), myeloid (CD13^+^), and B cell (CD19^+^) populations between the mock and TDX2 groups (Figure 5D). In the spleen, we evaluated T, B, myeloid, and stem cells populations. Equivalent populations were observed for all cell types between mock and TDX2 groups apart from the T cell population, where there was a slight elevation in CD3^+^ and CD4^+^ cells (CD3^+^, p < 0.0001; CD4^+^, p = 0.03) in the TDX2 group (Figure 5E). Human cell engraftment in WBCs was low, making specific leucocyte populations difficult to evaluate accurately.

### Effective Biodistribution and Efficacy of the IMP in NSG Mice with No Vector Shedding or Toxicity

Evaluating IMP biodistribution in the body provides important insight into which organs are potential therapeutic targets and can also identify possible off target effects such as gene transfer to the reproductive organs. We evaluated distribution of transduced human cells in non-hematopoietic organs, including the liver, heart, lungs, kidney, brain, and gonads in addition to the spleen, thymus, WBCs, and BM. Biodistribution was measured in the TDX2 group (n = 18) by qPCR using a method that only detects integrated vector in human cells (primer-probe sets specific for integrated vector and human housekeeping gene). Importantly and consistent with the intended mode of action to treat MPSIIIA, transduced human cells were detected in the brain (12 out of 18). Transduced human cells were also identified in the BM (15/18), WBC (6/18), thymus (8/18), spleen (16/18), heart (7/18), liver (17/18), kidney (18/18), and lung (18/18) but undetectable in the gastrocnemius muscle (0/18) and also the gonads (0/18), suggesting no potential for germline transmission. The average VCN per human cell in the BM was 6.6, WBC 7.0, thymus 4.9, spleen 4.3, brain 6.7, heart 5.1, liver 3.6, kidney 4.6, and lung 4.1 of TDX2 animals (Figure 5F). The average VCN of all organs tested (both hematopoietic and non-hematopoietic) was equivalent to the VCN of transduced human cells (bulk liquid culture) prior to transplantation (5.2 versus 5.6, respectively) (Figure 4C). Efficacy of treatment was confirmed by the significant increase in BM SGSH enzyme activity in the TDX2 group compared to the mock group (Figure 5G). This reflects the 4% of human cells in the background of normal mouse BM that naturally have low SGSH activity levels.

Since this is an *ex vivo* stem cell gene therapy technique, we did not expect to observe vector shedding from transplanted transduced cells. Indeed, p24 ELISA confirmed undetectable levels of capsid protein in the plasma and urine of treated mice (Table S2). For toxicology analysis, blood and BM smears and formalin fixed samples of brain, heart, kidneys, liver, lungs and bronchi, skeletal muscle, spleen, and testes or ovaries were sent for H&E staining and evaluation by Envigo. Hematology and histopathology findings reported no differences between mock- and TDX2-treated NSG mice (Figures S3 and S4).

### LV CD11b.SGSH Demonstrates Low Transformation Potential

**Figure 6:**
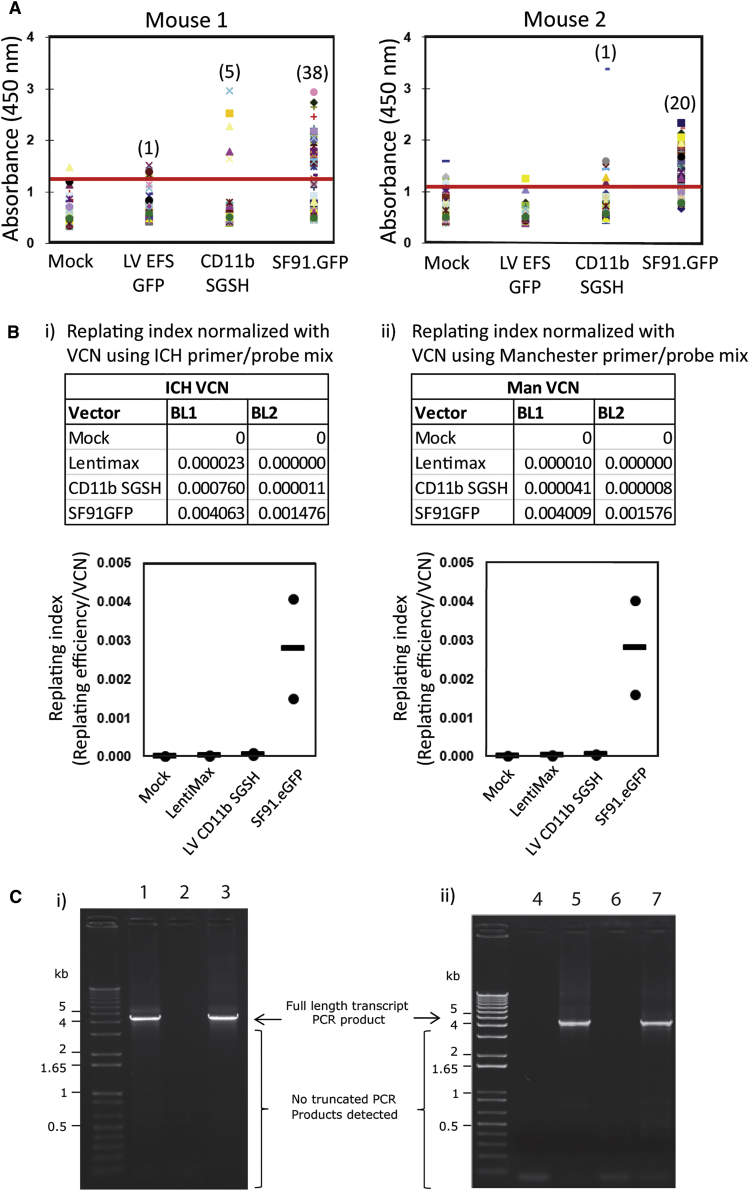
CD11b.SGSH LV Genotoxicity (A and B) *In vitro* immortalization (IVIM) assay. Murine Lin^−^ cells transduced by the indicated vectors were expanded as mass cultures for 2 weeks. An aliquot was taken for qPCR for VCN measurement. On day 15, cells were plated into 96-well plates at a density of 100 cells/well or 1,000 cells/well in 100 μL medium. Two weeks later, the wells showing abundant cell growth were counted as positive, and the frequency of replating cells was calculated based on Poisson statistics using L-Calc Software (StemCell Technologies, Vancouver, Canada). (A) WST-1 colorimetric proliferation assay to determine positive growth above baseline (red line) (n = 2). (B) Replating frequency corrected for VCN group by investigators at GOSH, UK (i) and University of Manchester (ii). Horizontal bars indicate mean values. (C) Cryptic splice site assessment. (i) 293T and (ii) CD34^+^ cells were transduced with LV-CD11b.SGSH at an MOI of 10. Seventy-two hours after transduction genomic DNA was isolated from cells and PCR performed to amplify the integrated transgene with primers upstream of CD11b and downstream of WPRE. (i) PCR products from LV-CD11b.SGSH transduced 293T cells (lane 1), non-transduced 293T cells (lane 2) and SGSH genome plasmid control (lane 3) were run on an electrophoresis gel. A strong band was observed at 4.1 kb indicating full-length transcript in LV-CD11b.SGSH transduced cells. No other prominent bands were detected, suggesting an absence of splice mutants. The same finding was observed in LV-CD11b.SGSH-transduced CD34^+^ cells (ii) (lane 4, non-template control; lane 5, SGSH plasmid control; lane 6, non-transduced control; and lane 7, LV-CD11b.SGSH transduced).

A long-term concern regarding the clinical use of lentiviral vectors is the risk of insertional mutagenesis. The *in vitro* immortalization (IVIM) assay has proved to be an effective tool to evaluate genotoxicity by determining transformation events in virally transduced murine lineage negative stem and progenitor cells under myeloid differentiation conditions.^8^, ^24^ We evaluated CD11b.SGSH vector against a positive control gamma-retroviral vector, gRVSF91GFP, which has an intact LTR comprising of the promoter and enhancer element of spleen focus-forming virus known to cause transformation events. A further control of a SIN LV with an internal SFFV promoter driving GFP (LV EFS GFP [lentimax]) was also included. The gRVSF91GFP-positive control vector generated high levels of transformation and cell proliferation, with markedly lower levels observed for the CD11b.SGSH vector with only a slight elevation in transformation events compared to lentimax negative control, although this was not significantly different (Figure 6A). The replating index (replating efficiency/VCN), which provides a relative measure of transformation potential, was determined separately at two institutions and confirmed the low transformation ability of the CD11b.SGSH vector (Figure 6B, i and ii).

Cryptic splice sites can cause pre-genome splicing during vector production, leading to generation of defective transgenes. In addition, aberrant splice products may encode protein variants that have immunogenic or toxic potential. To evaluate the presence of cryptic splice sites, human embryonic kidney 293T cells and human CD34^+^ cells were transduced with CD11b.SGSH LV and incubated for 72 h. Genomic DNA was extracted and PCR performed to evaluate the presence of full-length integrated transcripts. PCR primers were designed to bind upstream of the CD11b promoter and downstream of the woodchuck hepatitis virus (WHP) posttranscriptional regulatory element (WPRE) promoter to amplify a 4.1-kb region of integrated genome sequence. No prominent smaller bands were detected after gel electrophoresis of the PCR product, suggesting absence of any truncated transcripts (Figure 6C). This confirms incidence of splice-truncated vector is negligible with CD11b.SGSH LV.

## Discussion

Pre-clinical safety and efficacy studies are of paramount importance for the translation of new gene therapies to the clinic. We have developed a potentially promising approach of lentiviral-vector-mediated HSC gene therapy for the treatment of MPSIIIA, where the neurological phenotype of the condition is targeted by cross-correction. In this work, we performed the necessary studies typically required by the regulatory agencies before a first-in-human clinical study can be initiated in MPSIIIA patients. We confirmed equivalence among LV batches, determined an optimal transduction strategy of human HSCs, effectively scaled up the transduction process, and evaluated the effect of cryopreservation on transduced cells. Furthermore, we demonstrated effective engraftment and appropriate biodistribution of the IMP in a humanized mouse model and confirmed low vector genotoxicity.

The level of SGSH expression is likely to strongly influence the degree of MPSIIIA disease correction, given that standard allogeneic stem cell transplant with wild-type SGSH expression is ineffective in correcting the clinical phenotype in both MPSIIIA animal models and patients.^25^, ^26^, ^27^ We therefore evaluated the appropriate SGSH LV MOI required to achieve efficacy in human CD34^+^ cells without adverse toxicity. We predict an effective target number of integrated vector copies to be between 2 and 5 per cell, based on other current HSCGT studies and clinical trials.^4^, ^22^ Following a single transduction (at MOI 100), the median VCN achievable from four independent CD34^+^ cell transduction experiments was 2.34, compared to 4.95 following a double transduction. Both values are within our target range. There is argument to use a higher VCN to boost SGSH activity levels, further enhancing the likelihood of therapeutic benefit in patients, particularly as we expect a minimal threshold level of activity of approximately 8.5% normal levels required to correct brain neuropathology as previously determined.^6^, ^21^ MPSIIIA patients have a poor prognosis, with no effective treatments currently available and the potential benefit of SGSH overexpression is important. Although there is no evidence that increased vector copies lead to a higher risk of insertional activation, it is likely that in future studies, regulatory agencies may ask for an upper limit on VCN, hence our plan to target a limit of five vector copies. Ideally, we would have evaluated transduction of MPSIIIA patient CD34^+^ cells; however, to date we have been unable to source any MPSIIIA patient HSCs. Encouragingly, murine MPSIIIA HSCs transduced equally as well as WT, so we do not expect MPSIIIA HSC transduction to prove problematic.^21^

The equivalence study findings demonstrate that SGSH LV manufactured at GMP clinical grade maintains the ability to transduce and overexpress SGSH enzyme in both lineage-depleted MPSIIIA murine HSCs and human HSCs. Given these results, a simple comparison study in CD34^+^ cells will likely be sufficient to demonstrate consistency between vector batches prior to clinical use. In our studies, we observed variability in LV transduction efficiency between different batches of HSCs, an important consideration when devising the treatment strategy for patients. Notably the fold activity of SGSH per VCN was higher in human CD34^+^ cells than in murine lin^−^ cells. This reflects a 2- to 3-fold lower baseline WT/normal level of SGSH in human cells and a higher percentage of cells transduced—typically 80%–85% in hCD34^+^ cells versus 70%–80% in murine lin^−^ cells.

We effectively performed a large-scale transduction validation run, mimicking the CD34^+^ cell numbers, and expected LV dosing requirements similar to those that will be utilized in the clinical setting. Since patients will require time to receive full myeloablative treatment prior to transplant, we also evaluated the feasibility of cryopreserving transduced cells. We demonstrated that cryopreservation of transduced CD34^+^ cells for ≥ 52 days has a negligible influence on pharmacodynamic effects, as measured by enzyme activity, and CD34^+^ cell viability. Both VCN and SGSH activity were comparable in transduced cells before and after cryopreservation. We did observe up to 20% cell loss after thawing, which will need to be accounted for when calculating cell doses for transplant. The CFU assay performed post-cryopreservation of the large-scale double-transduced product showed a reduction in cell recovery of approximately 33% compared to the single-transduced product, a finding not observed at small scale in non-cryopreserved cells but that will, however, need to be considered when finalizing patient gene modified cell-dosing strategy for the clinic.

We utilized the humanized NSG mouse model to evaluate the effectiveness of SGSH LV-transduced HSC engraftment, identify which organs they distribute to, and evaluate any toxicological effects or potential for vector shedding. We adopted a dosing protocol that mimics the proposed clinical protocol, using intravenous administration with the appropriate safety margins, i.e., assessing the highest expected treatment dose (MOI 100 and double-transduced cells). Mice were not extended out beyond 16 weeks due in part to the high prevalence of spontaneous thymic lymphomas in older NSG mice and increased incidences of premature death, which would make interpretation of treatment-related histopathological findings difficult.^28^ NSG mice can only tolerate a partial myeloablative dose of busulfan; as such, HSC engraftment is not as high as observed in MPSIIIA mice that can tolerate a full conditioning regime. Furthermore, HSCs sourced from PBMC do not engraft as effectively as UCB sourced HSCs in NSG mice; however, the cell source of choice for patients will be their own PBMCs. Nonetheless, we were able to demonstrate successful engraftment of double-transduced and cryopreserved PBMC-derived CD34^+^ cells in the hematopoietic organs of transplanted NSG mice at comparable levels to mock-transduced cells. Furthermore, the pattern of engraftment we observed was comparable to similar studies conducted by Visigalli et al.^29^ to evaluate engraftment of iduronidase (IDUA) LV-transduced healthy donor HSPCs for MPSI and by Carbonaro et al.^8^ evaluating pre-clinical safety of HSCGT for ADA-SCID, both of which utilized UCB-CD34^+^ cells as their donor source without any cryopreservation. Our findings demonstrate that cryopreservation of the IMP is a viable strategy for future autologous hematopoietic stem cell gene therapies.

Finally, to assess the insertional mutagenesis potential of the SGSH LV, we used the *in vitro* platform established by Modlich et al.^24^ Low incidence of transformation events in the CD11b driven SGSH LV demonstrates the relative safety of these SIN design vectors with their internal mammalian promoters and was comparable to other SIN vectors currently used in the clinic.^8^, ^30^ Integration site analysis in human cells will be evaluated as one of the safety parameters during phase I-II clinical trial.

Taken together, these studies demonstrate that genetic modification of CD34^+^ cells using CD11b.SGSH LV fulfills the essential pre-clinical safety and efficacy requirements prior to commencing a phase I-II HSCGT clinical trial in MPSIIIA patients.

## Materials and Methods

### Manufacture and Titer of Lentiviral Vector

Research grade pCCLsin.hCD11b.SGSH.ΔWPRE (CD11b.SGSH LV) was produced and titer determined as previously described.^6^, ^21^ Clinical grade CD11b.SGSH LV was produced under GMP at King’s College London Vector production facility and quality control (QC) testing prior to vector release was performed by Bioreliance (UK). The infectious titer for GMP vector was determined on a human HT29 cell line with serial dilutions of vector, calculating the integrated vector by validated qPCR assay at University College London as previously described.^8^

### Experimental Animals

All *in vivo* procedures were ethically approved under UK Home Office regulations. Mice were housed in a 12-/12-h light/dark cycle with food and water provided *ad libitum*.

#### MPSIIIA Mice

MPSIIIA mice^31^ on a C57BL/6J background (B6.Cg-Sgshmps3a/6J) were maintained by heterozygote breeding.^6^, ^21^, ^32^, ^33^ MPSIIIA backcrossed to PEP3 CD45.1 (B6.SJL-Ptprca Pepcb/BoyJ) were used to distinguish donor and recipient cells as previously described.^6^ WT littermate controls were used throughout.

#### NSG Mice

NSG mice were obtained from The Jackson Laboratory (USA) and bred in-house.^34^

#### C57BL/6 Mice

For IVIM assays at University of Manchester and University College London, 7-week-old C57BL/6 inbred mice were purchased from Harlan Labs (UK).

### Isolation, Transduction, and Transplantation of Murine MPSIIIA BM lin^−^ HSCs

Donor BM cells were harvested by flushing tibias, femurs, and the pelvis of age-matched MPSIIIA/PEP3 (CD45.1+ve) mice. Isolated cells were then lineage depleted and hematopoietic stem cells transduced with GMP or non-GMP LV at an MOI of 60 as described previously.^6^ Recipient mice at 2 months of age were treated with intraperitoneal 125 mg/kg busulfan (Busilvex; Pierre Fabre) over 5 days prior to tail-vein injection of 3 × 10^5^ LV-transduced hematopoietic stem cells. Hematopoietic stem cell engraftment was assessed in PB following staining with anti-mouse CD45.1-PE (donor) and CD45.2-FITC (recipient), CD3, CD11b, and CD19 antibodies (BD PharMingen) and analyzed on a BD FACS Canto II flow cytometer.

### CD34+ve Cell Isolation, Transductions, and CFU Assay

CD34^+^ cells were isolated from mobilized PB, BM (NHS REC), or umbilical cord blood (soured from ethically approved Anthony Nolan trust) by magnetic separation following the manufacturers protocol (Miltenyi Biotec, UK). In brief, human cord blood or human BM was diluted 1:1 with Hanks balanced salt solution and layered over Ficoll Histopaque 1077 (Sigma) in 50 mL centrifuge tubes and centrifuged (no brake) at 480 × *g* for 30 min at room temperature. The mononuclear cells (the buffy coat) were harvested and CD34^+^ cells were isolated by immunomagnetic separation with the Miltenyi MACS CD34^+^ cell isolation kit (Miltenyi Biotech, UK). Cells were counted and either transduced as freshly isolated CD34^+^ cells or cryopreserved (freezing medium, 90% serum and 10% DMSO) and then transduced after thawing. CD34^+^ cells were seeded in growth medium (x-vivo 15 + 1% human serum albumin, 20 ng/mL recombinant human-interleukin-3 [rh-IL-3], 300 ng/mL rh-stem cell factor [rh-SCF], 300 ng/mL rh-fms-like tyrosine kinase 3 ligand [rh-FLT3L], and 100 ng/mL rh-thrombopoietin [rh-TPO]; Peprotech, UK) at a density of 0.5–1 × 10E6 cells/mL and pre-stimulated overnight for 18 ± 2 hrs. Cells were then transduced once (TDX1) with either GMP or non-GMP CD11b.SGSH LV at an MOI 100 for 16–18 hrs with cytokines. A proportion of these cells were seeded for CFU assay following manufacturer’s protocol (Stem Cell Technologies), while the remainder underwent a second round of transduction (TDX2) with either non-GMP or GMP CD11b.SGSH LV at an MOI 100 for a further 24 hrs prior to seeding in CFU assay.

#### Large-Scale Transduction and Cryopreservation

CD34^+^ cells were positively selected from two pooled PB mononuclear cell (PBMC) apheresis units (Allcells, USA) using the Miltenyi CliniMACSPlus instrument and reagents according to the manufacturer’s instructions. The selection yielded a total of 2.5 × 10^8^ viable cells, with a purity of 99.5% CD34^+^ confirmed by fluorescence-activated cell sorting (FACS). For pre-stimulation, cells were placed in growth media (X-VIVO 15 supplemented with 1% human serum albumin (HSA), IL-3 (20 ng/mL), SCF (300 ng/mL), FLT3-L (300 ng/mL), and TPO (100 ng/mL) (Peprotech) and seeded in a 118-C VueLife fluorinated ethylene propylene (FEP) culture bag at a density of 1 × 10^6^/mL. After approximately 22 hrs of pre-stimulation, cells were harvested, and a QC sample was taken for counting, viability, flow cytometry (CD34/CD45), and baseline control for VCN assay (VCN in bulk) and CFU assay (CFU counts). At that point cells were split into two arms: a transduction arm and a mock arm. Remaining cells were cryopreserved for downstream QC testing.

#### Transduction Arm

GMP SGSH-LV was added at an MOI of 100 (1 × 10^8^ TU/mL) to 60 × 10^6^ cells in growth media. The cells and vector mix (a total of 60 mL, cells at 1 × 10^6^/mL) was divided evenly between 2× 32-C bags and placed in the incubator for the first round of transduction (21 hrs). Following transduction, cells were harvested, pooled, and washed by centrifugation. After counts and viability, QC samples were distributed for flow cytometry (CD34/CD45), VCN assay (bulk only), and CFU assay (counts). A total of 60 × 10^6^ cells were set aside in growth media, and GMP SGSH-LV was added for a second round of transduction. After the second and final round of transduction, cells were harvested, washed by centrifugation, and re-suspended in X-VIVO 15/1% HSA. QC samples were taken for counts, viability, CD34/CD45 phenotype, VCN assay (bulk and picked CFU), CFU assay (counts), and SGSH activity (pooled CFUs).

#### Mock-Transduction Arm

45 × 10^6^ cells were set aside in growth media at a density of 1 × 10^6^/mL and transferred into a 45-C VueLife FEP culture bag for mock transduction 1. After 22.5 hr, cells were harvested, washed by centrifugation. After counts and viability, QC samples were distributed for flow cytometry (CD34/CD45), VCN assay (bulk only), and CFU assay (counts). 45 × 10^6^ cells were set aside in growth media at a density of 1 × 10^6^/mL and transferred into a 45-C VueLife FEP culture bag for mock transduction 2. Leftover cells were cryopreserved as described in section 4.3. After approximately 20 hrs, cells were harvested and washed by centrifugation. QC samples were taken for counts, viability, CD34/CD45 phenotype, VCN assay (bulk and picked CFU), CFU assay (counts), and SGSH activity (pooled CFUs).

#### Cryopreservation

Cells were cryopreserved in CryoStor 10 at 5 × 10^6^ cells/mL per vial using a control rate freezer (Planer Kryo 560).

### Transplantation of Human HSCs in NSG Mice

An equal number of male and female NSG mice (7–9 weeks old) were pre-conditioned with 25 mg/kg busulfan delivered by intraperitoneal (i.p.) injection 24 hr prior to transplant. Cryopreserved cells were thawed at 37°C and gently washed twice in RPMI media. Cells were administered intravenously by tail-vein injection at 4 × 10^5^ cells per mouse. The health of the mice was monitored daily and weights measured weekly throughout the study (Figure S3). Sixteen weeks post-transplant, mice were euthanized by i.p. injection with a lethal dose of pentobarbital (0.8 mL/kg). PB samples were collected by cardiac puncture, animals were perfused with PBS to eliminate any contaminating blood, and specified organs were harvested for downstream processing. Seven out of the fifty experimental mice were culled before the end of the study due to signs of sickness and ill health, not uncommon with immunocompromised NSG mice. A further three animals were excluded from the study due to low engraftment (<1% human cells in two or more organs). Of the remaining 40 animals, 22 were from the mock treatment group (10 f, 12 m) and 18 from the TDX2 treatment group (11 f, 7 m).

### VCN Analysis

For the murine MPSIIIA equivalence studies, the number of integrations was determined by qPCR using the WPRE and rodent GAPDH primer probe sets described,^21^ and a standard curve generated by serial dilution of DNA sample derived from an EL4 cell line clone (ALS EL4 eGFP 2.2) containing two copies of integrated WPRE gene/cell. For determination of VCN in human cells, qPCR was performed using the vector-specific WPRE primer-probe set^21^ and a human syndecan 4 gene (SDC4) specific primer-probe set (forward primer 5′-cagggtctgggagccaagt-3′, reverse primer 5′-gcacagtgctggacattgaca-3′, probe 5′-VIC-cccaccgaacccaagaaactagaggagaat-TAMRA-3′). A standard curve was generated by serial dilution of genomic DNA (gDNA) sample derived from a human HTC116 cell line clone containing three copies of integrated WPRE gene/cell kindly provided by Genethon, France.

### SGSH Activity

Protein was isolated from tissue and cell samples by sonication 3 × 5 s at 5 μm amplitude on ice. Lysates were centrifuged at 2,045 × *g* for 15 min at 4°C. A bicinchoninic acid (BCA) assay was performed on supernatants to determine total protein concentration (Pierce/Thermo UK). A total of 10 μg was loaded in the SGSH assay for organs and 1–5 μg for BM and pooled CFU colony samples. The SGSH enzyme activity was performed as previously described.^6^, ^21^, ^35^ In brief, the assay measures levels of 4-methylumbelliferone (4MU), a fluorescent product formed by two-step hydrolysis of the substrate 4-methylumbelliferyl-alpha-N-sulpho-D-glucosaminide. The sample SGSH activity desulfates the substrate over a 17-hr reaction at 47°C, followed by a secondary reaction with excess alpha glucosidase, releasing the fluorescent product, 4MU over 24 hrs at 37°C, which is detected by reading fluorescence at excitation (Ex) 360 nm/emission (Em) 450 nm. The SGSH activity is expressed as μM 4MU released/μg protein/17 hr or fold increase over non-transduced.

### P24 ELISA

Citrated plasma was collected from PB samples. An immune complex dissociation step was performed as described in the manufacturer’s protocol (R&D Systems). For positive controls, citrated plasma (from a mock-transduced NSG mouse) was spiked with un-concentrated lentiviral vector (CD11b.GFP LV titer ∼2 × 10^6^ TU/mL) and a serial dilution performed. P24 capsid was detected in the positive controls up to a dilution of ∼1 in 1,300 (Table S1). Urine was collected at time of scruffing prior to harvest and/or during organ collection. The amount of urine collected ranged from 2 to 90 μl for each mouse. The urine was made up to a total volume of 100 μl and ran in the assay.

### Flow Cytometry Analysis for Chimerism and Engraftment

Hematopoietic donor cell engraftment in MPSIIIA mice was assessed by FACS immunostaining of isolated WBCs with anti-mouse CD45.1-PE for donor and CD45.2-FITC for recipient hematopoietic cells (BD PharMingen, Oxford, UK) in a 5% solution of ToPro3 iodide (Molecular Probes, Paisley, UK) on a BD FACS Canto II flow cytometer. Immunophenotype was evaluated by immunostaining with anti-mouse CD3, CD11b and CD19 antibodies (BD PharMingen, Oxford, UK). The percentage of viable CD34^+^CD45^+^ cells was assessed by staining the cells with anti-CD34-PE and CD45-PerCP antibodies (Miltenyi Biotec, UK) with a 7-aminoactinomycin D (7-AAD) live versus dead stain (Thermo Fisher, UK). Human cell engraftment and immunophenotype in transplanted NSG mice were determined by FACS immunostaining. Cells were collected from the hematopoietic organs of transplanted NSG mice, blocked with Fc receptor (FcR) blocking reagent 1:100 (Miltenyi Biotec, UK) for 10 min at room temperature and then stained for the expression of the following surface markers: CD45-FITC, CD34-PE, CD13-APC, CD3-APC, CD4-PE, CD8-PerCP-vio700, CD19-PerCP-vio700 (Miltenyi Biotec, UK; all 1:11) and a NIR live versus dead cell stain (Thermo Fisher, UK). A percentage of CD45^+^ human cells > 1% were considered engrafted. Compensation was performed using Ultra compensation beads and the ArC Amine reactive compensation bead kit (Thermo Fisher, UK). Fluorescence minus one controls (FMOs) were included in order to evaluate flow cytometry data accurately. Samples were acquired on a BD FACS CANTO (Becton Dickinson), and FCS files were exported and analyzed with FlowJo software.

### Histopathology Analysis

#### MPSIIIA Mouse Toxicology

Brain, ear, esophagus, heart, kidneys, liver, lungs, gastrocnemius, lumbar spinal cord, spleen, stomach, testes, thymus, and trachea were fixed in 4% paraformaldehyde (PFA) overnight and stored in 70% EtOH before being outsourced to Envigo CRO for H&E staining and histopathology analysis along with methanol fixed blood films for blood cell examination. Samples were sent blinded and were processed at GLP (report FF54YX).

#### NSG Mouse Toxicology

Samples of brain, heart, kidney, liver, lungs and bronchi, ovaries, skeletal muscle, spleen, testes, and thymus were fixed in 10% neutral buffered formalin (Sigma, UK) and outsourced to Envigo for H&E staining and histopathology analysis along with methanol fixed blood and BM films for blood cell examination. Samples were sent blinded and were processed at GLP (report JC53CL).

### IVIM Assay

The IVIM assay was performed as previously described.^24^ In brief, cells were expanded after retroviral transduction as mass cultures for 2 weeks in Iscove’s modified Dulbecco’s medium containing 50 ng/mL mSCF, 100 ng/mL hFlt-3 ligand, 100 ng/mL hIL-11, 10 ng/mL mouse interleukin 3 (mIL-3), 10% fetal calf serum, 1% penicillin/streptomycin, and 2 mmol/L glutamine. Density was adjusted to 5 × 10E5 cells/mL every 3 days. After mass culture expansion for 14 days, cells were plated into 96-well plates at a density of 100 cells/well or 10 cells/well.^24^ Two weeks later, the positive wells were counted, and the frequency of replating cells was calculated based on Poisson statistics using L-Calc software (Stem Cell Technologies). Selected clones were expanded for further characterization.

### Cryptic Splice Site Assessment

HSCs and HEK293T cells were transduced overnight with CD11b.SGSH LV, and media refreshed and then grown for a further 72 h. Cells were harvested and gDNA eluted using the Genelute kit (Sigma, UK). PCR was performed to amplify integrated transcripts using forward primer 5′-AGAAGGAGAGAGATGGGTGCGAGAG-3′ designed to bind just after the HIV packaging signal and the reverse primer 5′-GAAGGAAGGTCCGCTGGATTGAGG-3′, which binds toward the end of the WPRE element. Gel electrophoresis was used to determine band sizes.

### Statistical Analysis

Statistical analysis was performed using GraphPad prism software (GraphPad Software, UK), analyzing datasets by two-way ANOVA, unless otherwise stated.

## Author Contributions

Conceptualization, B.W.B. and S.M.E.; Methodology, S.M.E. and B.W.B.; Validation, P.H.; Formal Analysis, P.H.; Investigation, S.M.E., A.L., S.W., J.T., A.S.Y., S.R., H.P., and M.A.; Resources, A.B., L.C., F.F., R.W., S.A.J., and H.B.G; Writing – Original Draft, S.M.E.; Writing – Review and Editing, S.M.E. and B.W.B.; Supervision, B.W.B. and S.M.E.

## Conflicts of Interest

At the time these studies were performed, H.B.G. was an employee of UCL Institute of Child Health before joining Orchard Therapeutics. B.W.B. holds the IP for the therapy described. B.W.B., R.W., S.A.J., A.B., and H.B.G. are shareholders and SAB members of Orchard Therapeutics. All other authors declared no conflict of interest.

## Supplemental Information

Document S1. Figures S1 and S2 and Tables S1–S4

Document S2. Article plus Supplemental Information
