# Supplementary material for: Pre-clinical Safety and Efficacy of Lentiviral Vector-Mediated Ex Vivo Stem Cell Gene Therapy for the Treatment of Mucopolysaccharidosis IIIA
Source: Mol Ther Methods Clin Dev. 2019 Apr 6;13:399–413. doi: 10.1016/j.omtm.2019.04.001 (PMC6479204; doi:10.1016/j.omtm.2019.04.001)
Supplement: Document S1. Figures S1 and S2 and Tables S1–S4 [file mmc1.pdf]

OMTM, Volume *13*

Supplemental Information

Pre-clinical Safety and Efficacy of Lentiviral Vector-Mediated *Ex Vivo* Stem Cell Gene Therapy for the Treatment of Mucopolysaccharidosis IIIA

Stuart M. Ellison, Aiyin Liao, Shaun Wood, Jessica Taylor, Amir Saam Youshani, Sam Rowlston, Helen Parker, Myriam Armant, Alessandra Biffi, Lucas Chan, Farzin Farzaneh, Rob Wynn, Simon A. Jones, Paul Heal, H. Bobby Gaspar, and Brian W. Bigger

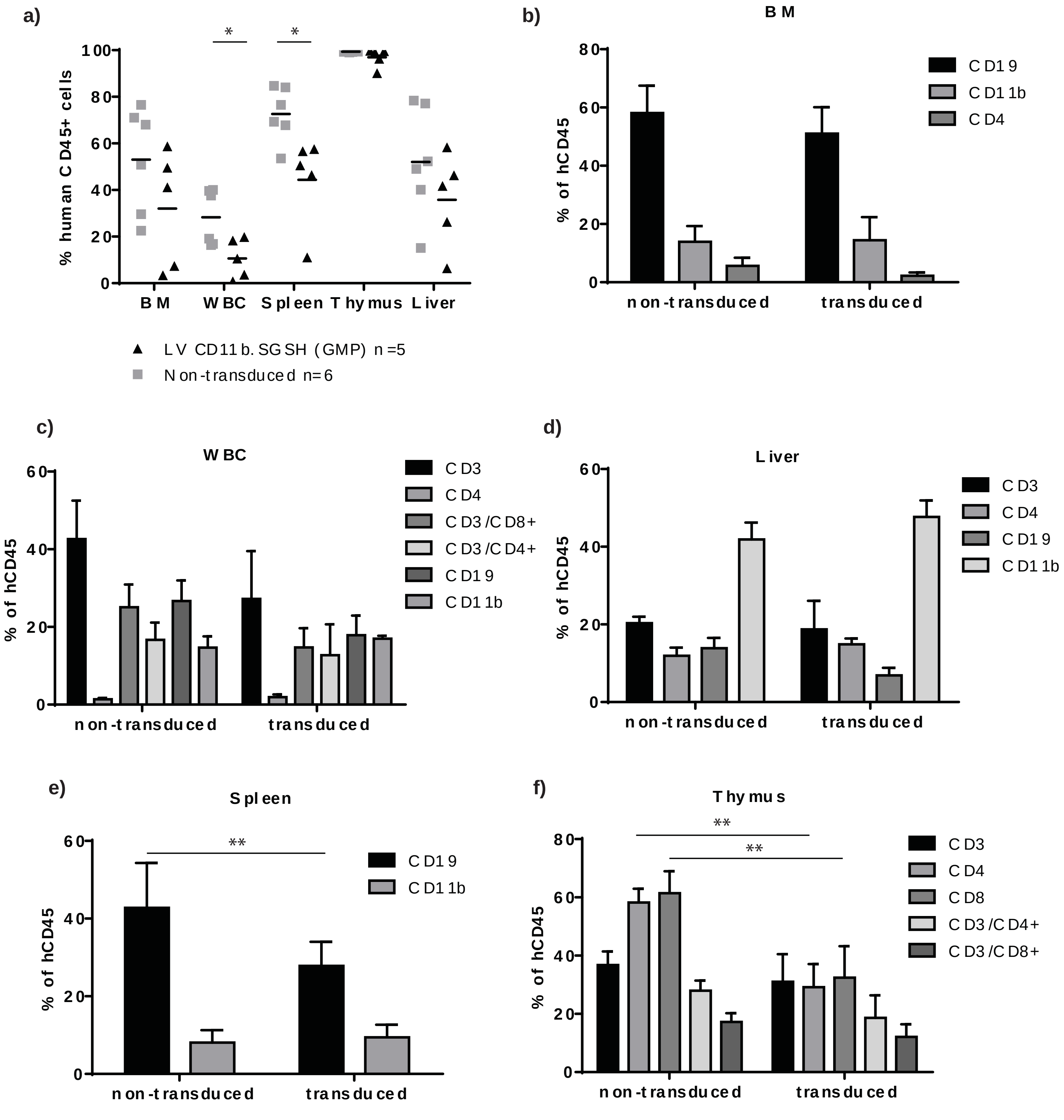

Supplementary Figure 1: UCB CD34+ cell engraftment in NSG mice. CD34+ cells isolated from umbilical cord blood (UCB) were thawed and pre-stimulated for 18hrs overnight (x-vivo 15 + 1%HSA 300ng/ml hSCF, 100ng/ml hTPO, 300ng/ml hFLT3 and 20ng/ml IL3). A single transduction with GMP grade CD11b.SGSH LV (KCL1) was performed at an MOI of 100 (EL4 titre) for 18hrs. A control group of non-transduced CD34+ cells was also included however they did not undergo the same manipulations/incubation times as transduced cells. NSG mice received 1x 25mg/kg dose of busulfan 24hrs prior to bone marrow transplant. Following transduction, cells were washed with x-vivo15 +1%HSA and then administered into the tail vein of partially myeloablated NSG mice at 3.4-3.7x105 cells per mouse. Mice were incubated for 16 weeks before harvesting hematopoietic organs for analysis by flow cytometry. Cells were isolated from peripheral blood (white blood cells (WBCs)), bone marrow, spleen, thymus and liver and stained for flowcytometry analysis.

a) Male body weights during study

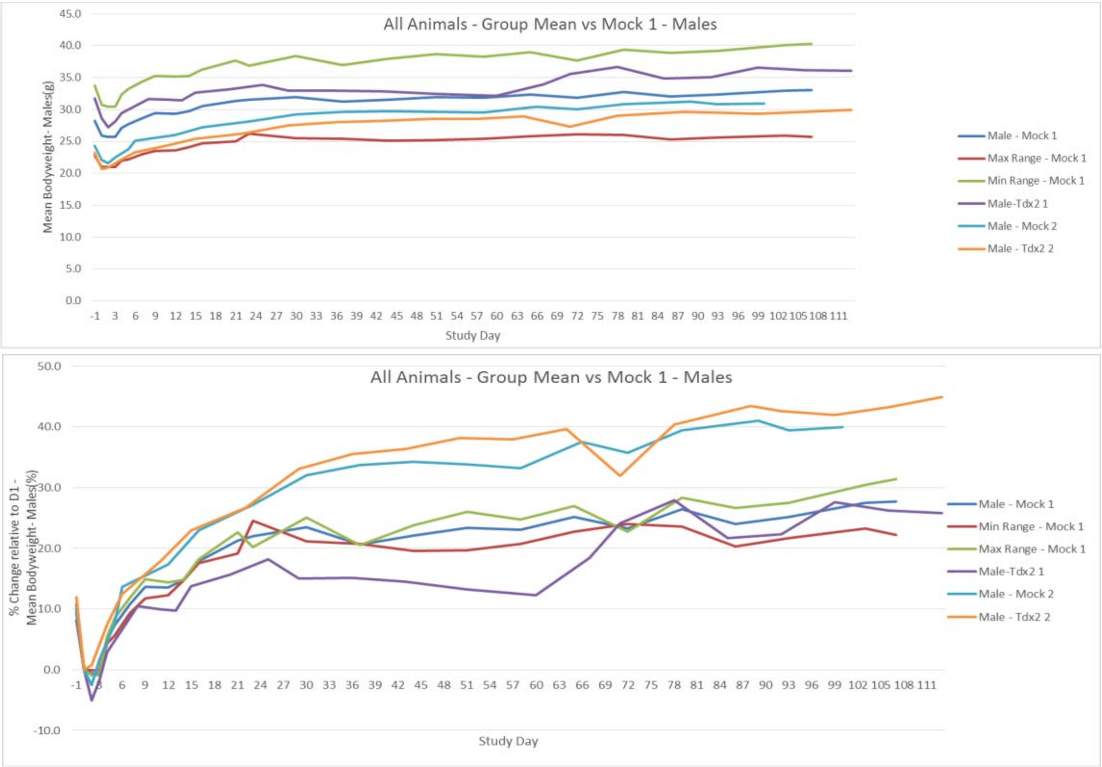

b) Female body weights during study

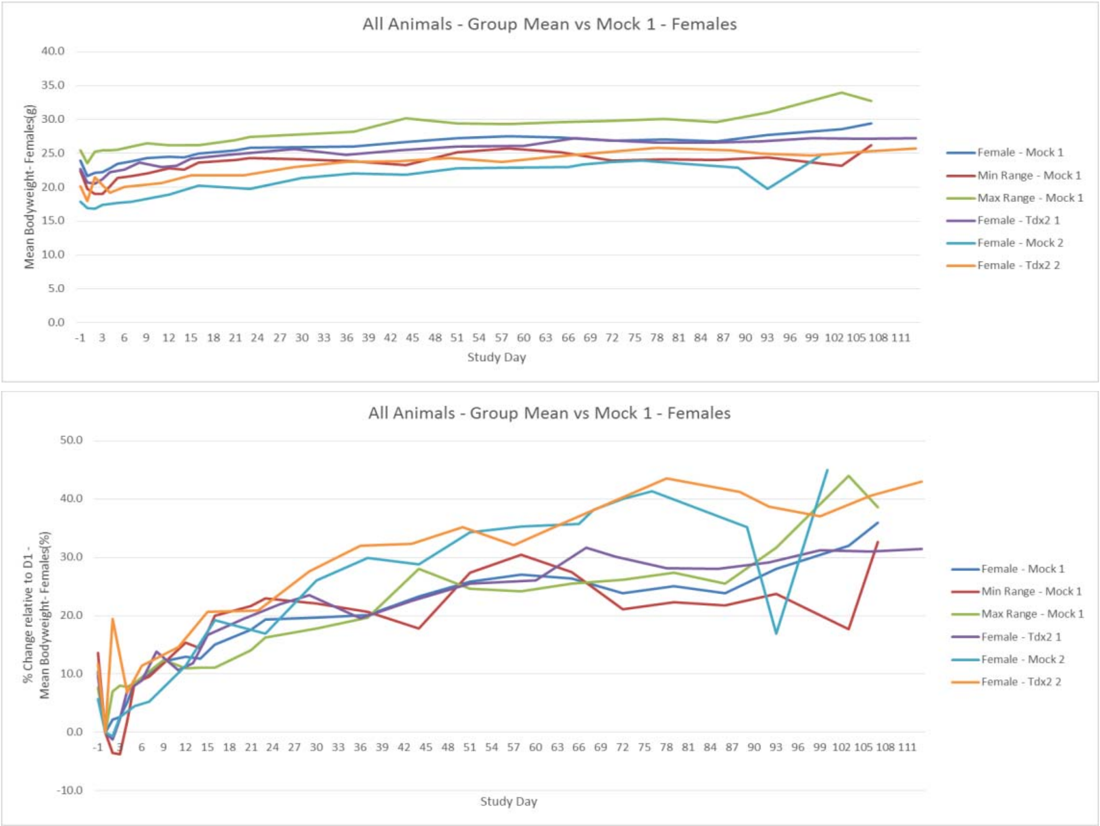

Supplementary Figure 2: NSG biodistribution study using mock or TDX2 transduced CD34+ cells from PBMCs in life data. The condition of NSG mice and their weights were monitored consistently throughout the study. Weights were evaluated every day for the first 2 weeks then weekly throughout the rest of the study for (a) male and (b) female treatment groups.

Supplementary Table 1 Histopathology – Group distribution of findings, MPSIIIAequivalence study

Request ID: 5161415

Group/sex	1M	2M	3M	4M
Model	WT	MPSIIIA	MPSIIIA	MPSIIIA
Treatment	0	0	Lab LV CD11b.SGSH	GMP LV CD11b.SGSH

		Number of animals affected			
	Group/Sex	1M	2M	3M	4M
Tissue/Organ and Findings	No. of animals	3	3	10	10
Brain	No. examined	3	3	10	10
Ear(s)/Pinna(e)	No. examined	3	3	10	10
Esophagus	No. examined	3	3	10	10
Heart	No. examined	3	3	10	10
Vacuolation, Myocardial	Minimal	0	1	4	3
	Slight	0	2	0	0
	Total	0	3	4	3
Kidneys	No. examined	3	3	10	10
Basophilia, Tubular	Minimal	1	0	0	0
	Total	1	0	0	0
Infiltrate, Inflammatory Cell	Minimal	1	0	2	1
	Slight	0	3	0	0
	Total	1	3	2	1

Supplementary Table 1: Histopathology findings from organs of MPSIIIA mice. Brain, ear, oesophagus, heart, kidneys, liver, lungs, gastrocnemius, lumbar spinal cord, spleen, stomach, testes, thymus and trachea were fixed in 4% PFA and 70% EtOH and outsourced to Envigo Ltd for H&E staining and histopathology analysis along with methanol fixed blood films for blood cell examination.

TABLE 1 (cont) Histopathology - group distribution of findings Request ID: 5161415

Group/sex	1M	2M	3M	4M
Model	WT	MPSIIIA	MPSIIIA	MPSIIIA
Treatment	0	0	Lab LV CD11b.SGSH	GMP LV CD11b.SGSH

		Number of animals affected			
	Group/Sex	1M	2M	3M	4M
Tissue/Organ and Findings	No. of animals	3	3	10	10
Kidneys	No. examined	3	3	10	10
Cast(s), Hyaline	Minimal	2	0	4	4
	Total	2	0	4	4
Infiltrate, Inflammatory Cell, Perivascular	Minimal	0	1	0	1
	Total	0	1	0	1
Dilatation, Tubular	Minimal	0	1	1	2
	Total	0	1	1	2
Vacuolation, Tubular Epithelium	Minimal	0	0	3	5
	Slight	0	3	2	0
	Total	0	3	5	5
Liver	No. examined	3	3	10	10
Vacuolation, Hepatocellular, Centrilobular	Moderate	1	0	0	0
	Total	1	0	0	0

TABLE 1 (cont) Histopathology - group distribution of findings Request ID: 5161415

Group/sex	1M	2M	3M	4M
Model	WT	MPSIIIA	MPSIIIA	MPSIIIA
Treatment	0	0	Lab LV CD11b.SGSH	GMP LV CD11b.SGSH

		Number of animals affected			
	Group/Sex	1M	2M	3M	4M
Tissue/Organ and Findings	No. of animals	3	3	10	10
Liver	No. examined	3	3	10	10
Infiltrate, Inflammatory Cell	Minimal	1	0	1	5
	Slight	0	1	0	1
	Total	1	1	1	6
Necrosis, Hepatocellular, Focal	Minimal	0	0	0	2
	Slight	0	0	0	1
	Total	0	0	0	3
Glycogen, Decreased	Total	0	0	3	4
Hypertrophy, Hepatocellular, Centrilobular	Minimal	0	0	0	3
	Total	0	0	0	3
Lungs and Bronchi	No. examined	3	3	10	10
Infiltrate, Inflammatory Cell, Perivascular	Minimal	0	0	0	1
	Slight	1	1	0	0
	Total	1	1	0	1

Group/sex	1M	2M	3M	4M
Model	WT	MPSIIIA	MPSIIIA	MPSIIIA
Treatment	0	0	Lab LV CD11b.SGSH	GMP LV CD11b.SGSH

Tissue/Organ and Findings	Group/Sex<br>No. of animals	Number of animals affected<br>1M<br>3	2M<br>3	3M<br>10	4M<br>10
Lungs and Bronchi	No. examined	3	3	10	10
Hemorrhage, Perivascular/Peribronchiolar	Slight	1	0	0	0
	Total	1	0	0	0
Cellularity, Increased, BALT	Minimal	0	1	0	0
	Slight	0	0	1	0
	Total	0	1	1	0
Erosion, Bronchial/Bronchiolar Epithelium	Slight	0	0	1	0
	Total	0	0	1	0
Skeletal muscle, Gastrocnemius	No. examined	3	3	10	10
Spinal Cord Lumbar	No. examined	3	3	7	10
Spleen	No. examined	3	3	10	10
Pigmented Macrophages, Increased	Slight	0	0	1	0
	Total	0	0	1	0

TABLE 1 (cont) Histopathology - group distribution of findings Request ID: 5161415

Group/sex	1M	2M	3M	4M
Model	WT	MPSIIIA	MPSIIIA	MPSIIIA
Treatment	0	0	Lab LV CD11b.SGSH	GMP LV CD11b.SGSH

		Number of animals affected			
	Group/Sex	1M	2M	3M	4M
Tissue/Organ and Findings	No. of animals	3	3	10	10
Stomach	No. examined	3	3	10	10
Infiltrate, Inflammatory Cell, Mucosal/Submucosal, Glandular Region	Minimal	0	1	1	2
	Slight	0	0	1	0
	Total	0	1	2	2
Dilatation, Glands	Minimal	0	0	0	1
	Total	0	0	0	1
Testes	No. examined	3	3	10	10
Vacuolation, Tubular	Minimal	1	0	0	0
	Slight	0	0	1	0
	Moderate	0	0	2	1
	Marked	0	0	7	9
	Total	1	0	10	10
Degeneration/Atrophy, Tubular	Severe	0	0	10	10
	Total	0	0	10	10

TABLE 1 (cont) Histopathology - group distribution of findings

Group/sex	1M	2M	3M	4M
Model	WT	MPSIIIA	MPSIIIA	MPSIIIA
Treatment	0	0	Lab LV CD11b.SGSH	GMP LV CD11b.SGSH

		Number of animals affected			
	Group/Sex	1M	2M	3M	4M
Tissue/Organ and Findings	No. of animals	3	3	10	10
Testes	No. examined	3	3	10	10
Infiltrate, Inflammatory Cell	Minimal	0	0	1	0
	Total	0	0	1	0
Thymus	No. examined	3	3	9	10
Trachea	No. examined	3	3	10	10

	pg/ml p24		
**Sample**	**Controls**	**Plasma**	**Urine**
**U1**	> LD		
**C0**	> LD		
**C1**	> LD		
**C2**	534.29		
**C3**	38.71		
**C4**	7.65		
**C5**	1.9 (< LD)		
**C6**	0		
**5LL**		0.00	1.50
**6L**		0.00	0.46
**6R**		0.00	0.81
**6B**		0.46	1.27
**6RR**		0.58	0.35
**7L**		0.00	1.15
**7R**		1.61	0.69
**7B**		0.12	0.69
**7LL**		0.00	no sample
**11L**		0.00	1.61
**11R**		2.88	1.15
**11B**		0.00	0.00
**12L**		0.46	1.38
**12B**		0.00	1.15
**12LLR**		0.00	1.84
**13L**		0.00	0.35
**13R**		0.92	0.81
**13LL**		0.00	no sample

Standards	pg/ml p24
**S1**	500
**S2**	250
**S3**	125
**S4**	62.5
**S5**	31.3
**S6**	15.6
**S7**	7.81
**S8**	0

Controls*	LV transducing units
**U1**	1000
**C0**	10000
**C1**	3333
**C2**	556
**C3**	93
**C4**	15
**C5**	1
**C6**	0

U = urine
C = citrated plasma (from Mock NSG mouse)

*spiked with LV

Supplementary table 2: No vector shedding detected in plasma or urine of transplanted NSG mice. A p24 ELISA was performed on blood and urine samples from TDX2 transplanted NSG mice to investigate potential for vector shedding following HSCGT. A number of controls were including consisting of either urine or citrated plasma from mock-transduced NSG mice spiked with varying amounts of control LV. The minimum detectable dose (MDD) specified in the HIV-1 Gag p24 ELISA kit (R&D systems) is 3.25pg/ml. All study samples tested were below the MDD. Plasma and urine control samples containing 15 LV transducing units or more gave a positive p24 signal.

Supplementary Table 3 – Histopathology findings from organs of NSG engraftment/bio-distribution study

TABLE 3 **Histopathology - group distribution of findings**

Dose Group	1	2
Treatment (cell line)	Non-transduced CD34+	Transduced CD34+

Tissue/Organ and Findings	Group/Sex<br>No. of animals	1M<br>12	2M<br>7	1F<br>10	2F<br>11
		Number of animals affected			
Brain	No. examined	12	7	10	11
Heart	No. examined	12	7	10	11
Kidneys	No. examined	12	7	10	11
Cast(s), Hyaline	Minimal	3	0	4	7
	Total	3	0	4	7
Vacuolation, Tubular Epithelium	Minimal	8	5	10	6
	Moderate	0	0	0	1
	Total	8	5	10	7
Dilatation, Pelvic	Slight	0	0	0	1
	Total	0	0	0	1
Basophilia, Tubular	Minimal	1	2	1	1
	Total	1	2	1	1

Supplementary Table 3 and 4: Histopathology findings from organs of NSG engraftment/biodistribution study. Sections of brain, heart, kidney, liver, lungs and bronchi, ovaries, skeletal muscle, spleen, testes and thymus were fixed in 10% neutral buffered formalin were outsourced to Envigo Ltd for H&E staining and histopathology analysis along with methanol fixed blood and bone marrow films for blood cell examination.

TABLE 3 (cont) Histopathology - group distribution of findings Request ID: 5185868

Dose Group	1	2
Treatment (cell line)	Non-transduced CD34+	Transduced CD34+

Tissue/Organ and Findings	Group/Sex<br>No. of animals	1M<br>12	2M<br>7	1F<br>10	2F<br>11
		Number of animals affected			
Liver	No. examined	12	7	10	11
Infiltrate, Inflammatory Cell	Minimal	1	0	0	1
	Total	1	0	0	1
Hypertrophy, Hepatocellular, Centrilobular	Minimal	5	2	1	0
	Total	5	2	1	0
Mineralization	Minimal	0	1	0	0
	Total	0	1	0	0
Lungs and Bronchi	No. examined	12	7	10	11
Alveolar Macrophages, Foamy	Minimal	5	0	4	4
	Slight	0	1	0	0
	Total	5	1	4	4
Infiltrate, Inflammatory Cell	Minimal	1	0	0	0
	Total	1	0	0	0

TABLE 3 (cont) Histopathology - group distribution of findings

Dose Group	1	2
Treatment (cell line)	Non-transduced CD34+	Transduced CD34+

		Number of animals affected			
	Group/Sex	1M	2M	1F	2F
Tissue/Organ and Findings	No. of animals	12	7	10	11
Lungs and Bronchi	No. examined	12	7	10	11
Granuloma, Cholesterol	Minimal	0	1	1	1
	Total	0	1	1	1
Inflammation, Alveoli	Minimal	0	0	2	0
	Total	0	0	2	0
Ovaries	No. examined	-	-	10	11
Corpora Lutea, Absent	Total	-	-	2	5
Cyst(s), Hemorrhagic	Total	-	-	0	1
Cyst(s)	Total	-	-	1	0
Skeletal Muscle	No. examined	12	7	10	11
Degeneration, Myofiber	Minimal	0	0	1	0
	Total	0	0	1	0

TABLE 3 (cont) Histopathology - group distribution of findings

Dose Group	1	2
Treatment (cell line)	Non-transduced CD34+	Transduced CD34+

		Number of animals affected			
	Group/Sex	1M	2M	1F	2F
Tissue/Organ and Findings	No. of animals	12	7	10	11
Spleen	No. examined	12	7	9	11
Extramedullary Hemopoiesis, Increased	Minimal	2	0	3	0
	Slight	10	7	4	11
	Total	12	7	7	11
Testes	No. examined	12	7	-	-
Mineralization	Minimal	4	4	-	-
	Total	4	4	-	-
Degeneration/Atrophy, Tubular	Severe	12	7	-	-
	Total	12	7	-	-
Thymus	No. examined	11	7	9	8

Supplementary Table 4. Hematology– Group distribution of findings from blood/BM smears of NSG engraftment/bio-distribution study

TABLE 4a Hematology - group distribution of findings

Dose Group	1	2
Treatment (cell line)	Non-transduced CD34+	Transduced CD34+

		Number of animals affected			
	Group/Sex	1M	2M	1F	2F
Blood film examination	No. of animals	12	7	10	11
White blood cell differential	No. examined	8	6	4	10
	Mean value	73	71	74	79
Neutrophils	No. examined	8	6	4	10
	Total % Mean	79	80	74	76
Lymphocytes	No. examined	8	6	4	10
	Total % Mean	18	17	23	23
Monocytes	No. examined	8	6	4	10
	Total % Mean	3	2	4	1
Eosinophils	No. examined	8	6	4	10
	Total % Mean	1	1	0	0
Basophils	No. examined	8	6	4	10
	Total % Mean	0	0	0	0

All cell counts and mean values presented calculated excluding individual animals with white blood cell differential counts below 30

TABLE 4b Bone marrow - group distribution of findings

Dose Group	1	2
Treatment (cell line)	Non-transduced CD34+	Transduced CD34+

Marrow film examination	Group/Sex<br>No. of animals	1M<br>12	2M<br>7	1F<br>10	2F<br>11
		Number of animals affected			
Erythroid hyperplasia	No. examined	10	7	8	7
	Total	4	3	4	3
Fatty, reduced cellularity	No. examined	10	7	8	7
	Total	0	0	1	1

All bone marrow data presented as a comparison to animal 3(3L, MOCK)
Data excluding samples not supplied or unsuitable for evaluation
